# A Betulinic Acid Derivative, BA5, Induces G0/G1 Cell Arrest, Apoptosis Like-Death, and Morphological Alterations in *Leishmania sp*

**DOI:** 10.3389/fphar.2022.846123

**Published:** 2022-03-22

**Authors:** Tatiana Barbosa dos Santos Magalhães, Dahara Keyse Carvalho Silva, Jessica da Silva Teixeira, Juliana Dizaira Teles De Lima, José Maria Barbosa-Filho, Diogo Rodrigo Magalhães Moreira, Elisalva Teixeira Guimarães, Milena Botelho Pereira Soares

**Affiliations:** ^1^ Laboratório de Histotécnica e Cultura Celular, Departamento de Ciências da Vida, Universidade Do Estado da Bahia (UNEB), Salvador, Brazil; ^2^ Laboratório de Engenharia Tecidual e Imunofarmacologia, Instituto Gonçalo Moniz, Fundação Oswaldo Cruz (FIOCRUZ), Salvador, Brazil; ^3^ Laboratório de Tecnologia Farmacêutica, Universidade Federal da Paraíba, João Pessoa, Brazil; ^4^ Instituto Senai de Inovação Em Sistemas Avançados Em Saúde, SENAI/CIMATEC, Salvador, Brazil

**Keywords:** leishmaniasis, betulinic acid, antileishmanial drugs, mechanism action, *L. amazonensis*

## Abstract

Leishmaniasis are endemic diseases caused by different species of intracellular parasites of the genus *Leishmania*. Due to the high toxicity and drug resistance of current antileishmanial drugs, it is necessary to identify new and more effective drugs. Previously, we investigated the immunomodulatory and anti-*Trypanosoma cruzi* action of BA5, a derivative of betulinic acid. In the present study, we investigated the *in vitro* activity of BA5 against different species of *Leishmania* and their action mechanism. BA5 exhibited low cytotoxicity against macrophages and inhibited the proliferation of promastigote forms of *Leishmania amazonensis* (IC_50_ = 4.5 ± 1.1 μM), *Leishmania major* (IC_50_ = 3.0 ± 0.8 μM), *Leishmania braziliensis* (IC_50_ = 0.9 ± 1.1 μM) and *Leishmania infantum* (IC_50_ = 0.15 ± 0.05 μM). Incubation with BA5 reduced the percentage of *Leishmania amazonensis*-infected macrophages and the number of intracellular parasites (IC_50_ = 4.1 ± 0.7 μM). To understand the mechanism of action underlying BA5 antileishmanial activity (incubation at IC_50_/2_,_ IC_50_ or 2xIC_50_ values of the drug), we investigated ultrastructural changes by scanning electron microscopy and evaluated cell cycle, membrane mitochondrial potential, and cell death against promastigote forms of *Leishmania amazonensis* by flow cytometry. Promastigotes incubated with BA5 presented membrane blebbing, flagella damage, increased size, and body deformation. Flow cytometry analysis showed that parasite death is mainly caused by apoptosis-like death, arrested cell cycle in G0/G1 phase and did not alter the membrane mitochondrial potential of *Leishmania amazonensis*. Surprisingly, the combination of BA5 and amphotericin B, an assay used to determine the degree of drug interaction, revealed synergistic effects (CI = 0.15 ± 0.09) on promastigotes forms of *Leishmania amazonensis*. In conclusion, BA5 compound is an effective and selective antileishmanial agent.

## Introduction

Leishmaniasis is a complex of diseases caused by different species of protozoa of the genus *Leishmania*. Despite being among the ten most relevant infectious diseases, leishmaniasis is part of a wide group of diseases worldwide neglected (Alvar et al., 2012; Silva et al., 2020). In 2021, WHO published that 54 countries are endemic to Visceral Leishmaniasis (VL) and 53 countries are endemic to Cutaneous Leishmaniasis (CL). Cases have been reported in about 98 countries, and 12 million people approximately have their lives affected by the different clinical spectra of the disease (WHO, 2021).

Clinical manifestations depend on factors inherent to the parasite, the natural resistance of the host and the magnitude of the immune response (Gabriel et al., 2019). After inoculation of promastigote forms through the bite of the insect vector, the parasite is internalized by host defense cells and differentiates into amastigote forms. In this way, the relationship between parasite and the host will determine the course of the disease. The host can be asymptomatic or develop classic skin and mucosal lesions, as well as atypical forms, such as diffuse and disseminated, as well as the visceral manifestation (Gupta et al., 2013; Gabriel et al., 2019). In the Americas, the main species that cause cutaneous leishmaniasis are *Leishmania amazonensis* and *Leishmania braziliensis* (Ministério da saúde do Brasil, 2017). On the other hand, visceral leishmaniasis is caused by *Leishmania infantum chagasi* in Brazil. The variability of species and their clinical outcomes are a challenge for the effective treatment and prophylaxis of the disease (Desjeux, 2004; Burza et al., 2018).

Although the knowledge of cell biology and immunology of leishmaniasis has advanced in recent decades, pharmacotherapy still lacks new alternatives. Pentavalent antimonial has been the first-line drugs since 1960, but they present several limitations such as high toxicity and adverse effects, resistance, the need for hospitalization and treatment failure. The second-line drugs, such as amphotericin B, pentamidine and miltefosine, also have several limitations, like high costs and teratogenicity (Romero and Lopez, 2017; Tiwari et al., 2018).

In this regard, the search for new active compounds plays an important role in the development of new antileishmanial drugs. Betulinic acid is a natural pentacyclic triterpene widely found in the plant kingdom. This compound has raised interest in the scientific community due to its vast number of biological activities, such as antitumor, anti-inflammatory, immunomodulatory, antimicrobial and antiparasitic activities (Takada and Aggarwal, 2003; Chen et al., 2008; Innocente et al., 2012; Sousa et al., 2014). Strategic structural changes of betulinic acid at position C-28 can generate more active molecules than its prototype (Yogeeswari and Sriram, 2005). In a previous study, we tested a series of semi-synthetic molecules derived from betulinic acid against *Trypanosoma cruzi*, and found compound BA5 active, causing ultrastructural changes in *T. cruzi*, such as loss of plasma membrane integrity and the appearance of atypical vacuoles, leading to death of the parasite by necrosis (Meira et al., 2016). Additionally, the immunomodulatory activity of BA5 was evaluated on macrophages and lymphocytes, being able to inhibit both the NF-kB and calcineurin pathways (Meira et al., 2017). In the present study, we evaluated the activity of BA5 *in vitro* against different species of *Leishmania*, and its mechanisms of action.

## Materials and Methods

### Drugs

Betulinic acid was extracted from the bark of *Ziziphus joazeiro* Mart., a native brazilian tree from the Rhamnaceae family, according to the methodology previously described (Barbosa-Filho et al., 1985). The semi-synthetic compound BA5 was prepared from betulinic acid as previously described (Barbosa-Filho et al., 1985) and used in antileishmanial assays (BA5; 94–98% purity by high performance liquid chromatography). Amphotericin B (Gibco Laboratories, Gaithersburg, MD) was used as positive control in antileishmanial assays. Gentian violet (Synth, São Paulo, SP, Brazil) was used as positive control in the cytotoxicity to mammalian cell assays. All compounds were dissolved in dimethyl sulfoxide (DMSO; PanReac, Barcelona, Spain) and diluted in cell culture medium for use in the assays. The final concentration of DMSO was less than 0.1% in all *in vitro* experiments.

### Animals

Male 4–6-weeks old BALB/c were used. All mice were raised and maintained at the animal facilities of the Gonçalo Moniz Institute, Oswaldo Cruz Foundation, Salvador, Brazil in sterilized cages, under a controlled environment and receiving a balanced rodent diet and water *ad libitum*. All experiments were approved by the local Animal Ethics Committee (Approval number: 004/2019).

### Parasites

*L. amazonensis* (MHOM/BR88/BA-125 Leila strain), *L. major* (MHOM/RI/WR173), *L. braziliensis* (MHOM/BR88/BA-3456) promastigotes were cultivated in Schneider (Sigma, St. Louis, MO, United States) medium supplemented with 10% fetal bovine serum (FBS) (Gibco) and 50 μg ml^−1^ Gentamicin (Sigma). *L. infantum* (MCAN/BR/89/BA262) promastigotes were cultivated in liver infusion tryptose (LIT) medium supplemented with 20% fetal bovine serum and 50 μg ml^−1^ Gentamicin, pH 7.2, at 26°C until logarithmic phase. Log phase promastigotes were used to study the effects of the betulinic acid and BA5 derivative.

### Viability Assay

*L. amazonensis, L. major, L. braziliensis* and *L. infantum chagasi* promastigotes (1 × 10^6^ cells/well) were incubated into 96-well plates, cultivated in Schneider (Sigma) medium supplemented with 10% fetal bovine serum (FBS) (Gibco) and μg mL^−1^ Gentamicin (Sigma). Drugs were added at six concentrations ranging from 1.56 to 50 µM in triplicate, and the plate was incubated for 72 h at 26°C. Amphotericin was added at eight concentrations ranging 0.0156–2.0 µM. Promastigotes viability was measured by twenty µL/well of AlamarBlue (Invitrogen, Carlsbad, CA, United States) during 2 h (*L. amazonensis, L. major and L. braziliensis*) or 24 h for *L. infantum* due to slower metabolism (Corral et al., 2013), after which colorimetric readings were performed at 570 and 600 nm.

### Cytotoxicity to Mammalian Cell

Peritoneal exudate macrophages were obtained by washing of the peritoneal cavity of BALB/c mice with cold Dulbecco’s Modified Eagle’s Medium (DMEM Life Technologies, GIBCO-BRL), 5 days after injection of 3% thioglycolate in saline (1.5 ml per mice). Cells were added into 96-well plates at a density 1 × 10^5^ cells/well containing DMEM medium supplemented with 10% of fetal bovine serum (FBS; Gibco) and 50 μg ml^−1^ Gentamicin (Novafarma, Anapolis, Brazil) and incubated for 24 h at 37°C and 5% CO_2_. Drugs was added in triplicate at eight concentrations ranging from 0.04 to 100 µM and incubated for 72 h. Twenty µL/well of AlamarBlue (Invitrogen) was added to the plates during 10 h. Colorimetric readings were performed at 570 and 600 nm. CC_50_ values were calculated using data-points gathered from three independent experiments. Gentian violet (Synth, Sao Paulo, Brazil) was used as positive control, at concentrations ranging from 0.04 to 10 µM.

### *In vitro* Macrophage Infection With *L. amazonensis*

Peritoneal exudate macrophages (5 × 10^5^ cells) were plated onto sterile coverslips in 24-well plates and kept for 24 h. The macrophages were infected with stationary growth phase promastigotes of *L. amazonensis* at a ratio of 10:1 macrophage at 35°C during 4 h and 5% CO_2_. Infected macrophages were incubated with different atoxic concentrations with values below the IC_50_ values of BA (9.4; 4.7; 2.3 µM) and BA5 (15.5; 7.7; 3.8 µM). After 24 h, the cells were fixed in methanol. The percentage of infected macrophages and the number of amastigotes/macrophages were determined by counting 100 cells per slides by counting the slides after Giemsa staining (Sigma) in an optical microscope (Olympus, Tokyo, Japan). Amphotericin B (Gibco) was used as a positive control in this assay.

### Annexin V and Propidium Iodide Staining

Promastigotes of *L. amazonensis* (10^6^ cells/well) were incubated in 24-well plates and incubated with BA and BA5 in different concentrations (IC_50_ or 2x IC_50_) for 24 h at 26°C. Parasites were labeled with propidium iodide (PI) and annexin V using the annexin V-fluorescein isothiocyanate (FITC) apoptosis detection kit (Sigma) according to the manufacturer’s instructions. The experiment was performed using a BD FACSCalibur flow cytometer (Becton Dickinson Biosciences, San Jose, CA, United States) by acquiring 10,000 events, and data were analyzed by BD software FlowJo v10 (Tree Star, Ashland, OR).

### Cell Cycle Analysis

Promastigotes of *L. amazonensis* (1 × 10^7^/well) were incubated with BA5 (9.0 and 4.5 μM) for 48 h. Parasites were washed with saline, centrifuged for 10 min at 252.0 g and diluted in the lysis solution containing PI (0.1% Triton X-100 and 2 μg ml−1 propidium iodide in PBS) in the absence of light at 37°C. After 30 min, the samples were acquired on a LSRFortessa flow cytometer (Becton Dickinson Biosciences, San Jose, CA, United States) and analyzed by FlowJo v.10 software (Tree Star).

### Analysis of Mitochondrial Membrane Potential

To determine the effect of the compound on mitochondrial membrane potential, *L. amazonensis* promastigotes were incubated with 9.0 and 4.5 μM of BA5 for 72 h. After the treatment, parasites were incubated with 10 μg/ml of rhodamine 123 (Sigma Aldrich, St. Louis, United States) for 15 min. Methanol was used as negative control. Data acquisition was performed using a LSRFortessa flow cytometer and the analysis was performed by FlowJo v.10 software.

### Scanning Microscopy Electronic

*L amazonensis* promastigotes (1 × 10^7^) were incubated with three concentrations from the IC_50_ values (2.25, 4.5 and 9.0 μM) of BA5 for 48 h at 26°C. The parasites were fixed in a 2% glutaraldehyde solution and 0.1 M sodium cacodylate buffer for 2 h at room temperature. After fixation, the cells were post-fixed in osmium tetroxide (1%) for 1 h at room temperature. The parasites were placed on glass cover slips with 0.01% poly-L-lysine, dehydrated in graded ethanol (30–100%) and submitted at critical point (replacement of ethanol by CO_2_) LEICA CPD 030. Samples were metalized with gold and observed in the scanning electron microscope JEOL JSM-6390LV.

### Drug Combination Assay

Isobolograms were constructed by the fixed ratio method. Serial double dilutions were performed in triplicate in ratios of 1:1 and 10:1, BA5 and amphotericin B, respectively, using *L. amazonensis* promastigotes. For each proportion, an IC_50_ value was calculated for each drug and combination after 72 h of incubation. The fractional inhibitory concentrations (FIC) were calculated by (IC_50_ when combined/IC_50_ isolated drug). The FIC values of different ratios were used to construct the isobologram in Graph Pad Prism version 5.01 program (Graph Pad Software, San Diego, CA, United States). The analysis of the combined effects was performed by determining the combination index (CI) as described previously by Chou and Talalay, 2005. CI values were used as cutoff to determine synergism.

### Statistical Analysis

One-way analysis of variance and Newman-Keuls multiple comparison tests were employed by using Graph Pad Prism version 5.01 (Graph Pad Software, San Diego, CA, United States). Differences were considered significant when the values were of *p* < 0.05.

## Results

### Cytotoxicity and Activity of BA5 Against Promastigote Forms

**FIGURE 1:**
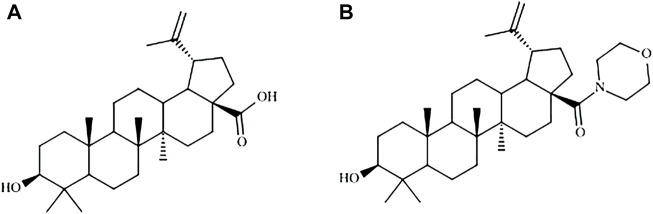
Molecular structures of betulinic acid **(A)** and BA5 derivative **(B)**.

Betulinic acid and BA5 derivative (Figure 1) presented CC_50_ values of 18.8 and 31.1 µM, respectively, to mammalian cells. Amphotericin B, the reference antileishmanial drug, presented a CC_50_ value of 3.3 µM, and gentian violet, a known cytotoxic drug, had a CC_50_ value of 0.5 μM (Table 1). The effect of BA5 on promastigote forms of different species of leishmania was evaluated at six different concentrations, ranging from 1.56 to 50 μM. As show in the Table 1, BA5 was effective against all tested species. After 72 h of incubation, BA5 inhibited *L. amazonensis* promastigote proliferation with an IC_50_ of 4.5 ± 1.1 μM; *L. major* (IC_50_ = 3.0 ± 0.8 μM), *L. braziliensis* (IC_50_ = 0.9 ± 1.1 μM) and *L. infantum* (IC_50_ 0.15 ± 0.05 μM). In addition, BA5 was 6.9 times more selective (IS) for *L. amazonensis* promastigotes, 10.4 times more selective for *L. major*, 34.5 more selective for *L. braziliensis* and 207 more selective for *L. infantum* when compared with mammalian cell*.* Furthermore, IS of BA5 was higher for *L. braziliensis* than amphotericin B. Betulinic acid exhibited little or no activity against promastigote forms of different species of leishmania. This prototype was not selective for *L. amazonensis* (IS = 0.66) and *L. braziliensis* (IS = 1.1) (Table 1).

**TABLE 1:** Cytotoxicity evaluation and antileishmanial activity against promastigotes of *L. amazonensis, L. major, L. braziliensis, and L. infantum*.

Compounds	Mammalian cells	Leishmania promastigotes							
	CC_50_ ± S.D. (μM)	IC_50_ ± S.D. (μM)	S.I.	IC_50_ ± S.D. (μM)	S.I.	IC_50_ ± S.D. (μM)	S.I.	IC_50_ ± S.D. (μM)	S.I.
	Macrophages	*L. amazonensis*		*L. major*		*L. braziliensis*		*L. infantum*	
BA	18.8 ± 0.1	29.2 ± 0.9	<1	>100	<1	16.3 ± 1.3	1.1	>100	<1
BA5	31.1 ± 1.2	4.5 ± 1.1	6.9	3.0 ± 0.8	10.4	0.9 ± 1.1	34.5	0.15 ± 0.05	207
Amphotericin B	3.3 ± 0.50	0.09 ± 0.02	36.6	0.2 ± 0.005	16.5	1.3 ± 0.09	2.5	0.0002 ± 0.0001	>1000
Gentian violet	0.3 ± 0.01	N.D.	N.D.	N.D.	N.D.	N.D.	N.D.	N.D.	N.D.

CC_50_, drug concentration that reduces cell viability by 50%; IC_50_, drug concentration that reduces the number of parasites by 50%. IC_50_ values for intracellular parasites were determined after 72 h. N.D., Not determined; S.D., Standard deviation; S.I., Selectivity Index. Values are means ± SD of three independent experiments performed in triplicate.

### BA5 Reduces the Infection of Macrophages by *L. amazonensis*

**FIGURE 2:**
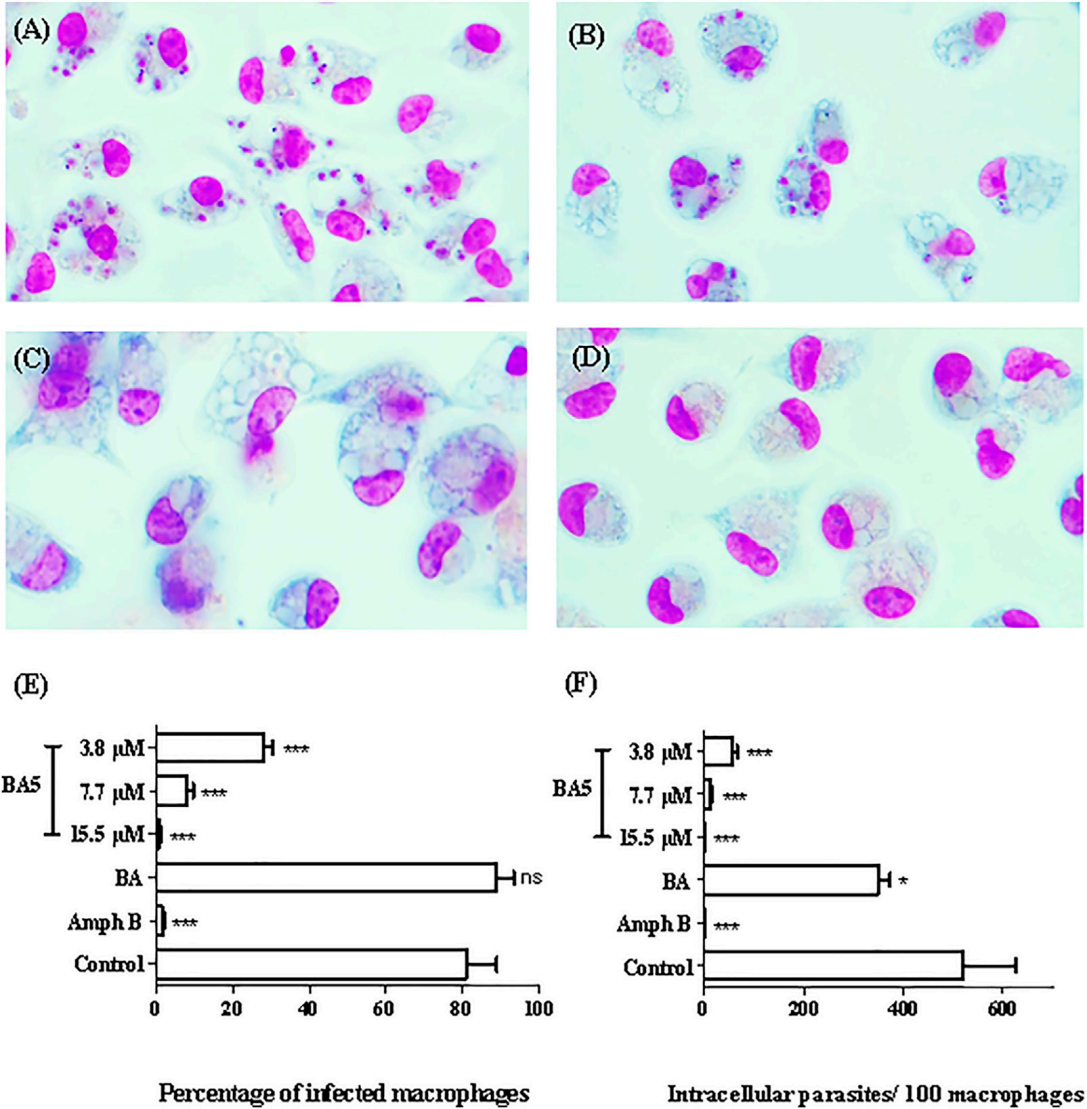
*In vitro* effects of BA and BA5 against intracellular parasites of *L. amazonensis*. Peritoneal macrophages of BALB/c mice were infected with promastigotes of *L. amazonensis* at stationary phase (10:1) and were treated with BA or BA5 for 24 h. **(A)** Untreated control. **(B)** Treatment with BA at 9.4 µM. **(C)** BA5 at 15.5 µM. **(D)** amphotericin B at 1.5 µM. 1000x magnification. The percentage of infection **(E)** and the number of intracellular parasites per 100 macrophages **(F)** were determined after 24 h of treatment. Amphotericin B was used as positive control. *p < 0.05; ***p < 0.001.

BA and BA5 promoted a significant decrease in the number of *L. amazonensis*-infected macrophages after 24 h of treatment (Figure 2). BA5 decreased the percentage of infected cells and the number of intracellular parasites at all concentrations tested, in a concentration-dependent manner (Figures 2E,F). BA prototype reduced the number of intracellular forms per macrophage only in the highest concentration tested, presented IC_50_ value greater than 200 and was not selective against the parasite (SI < 1) (Table 2). BA5 presented an IC_50_ value of 4.1 ± 0.7 µM (SI = 7.5) and amphotericin B exhibited an IC_50_ value of 0.05 ± 0.02 µM (SI = 66) (Table 2).

**TABLE 2:** Inhibitory concentration for 50% of intracellular parasites forms and selectivity index.

Compounds	*L. amazonensis* (intracellular parasites)	
	IC_50_	S.I. (µM)
BA	>200	<1
BA5	4.1 ± 0.7	7.5
Amphotericin B	0.05 ± 0.02	66.0

IC_50_ values for intracellular parasites were determined after 24 h. N.D., not determined; S.D., Standard deviation.; S.I., Selectivity Index. Values are means ± SD, of three independent experiments performed in triplicate.

### Ultrastructural Alterations in BA5-Treated Leishmania

**FIGURE 3:**
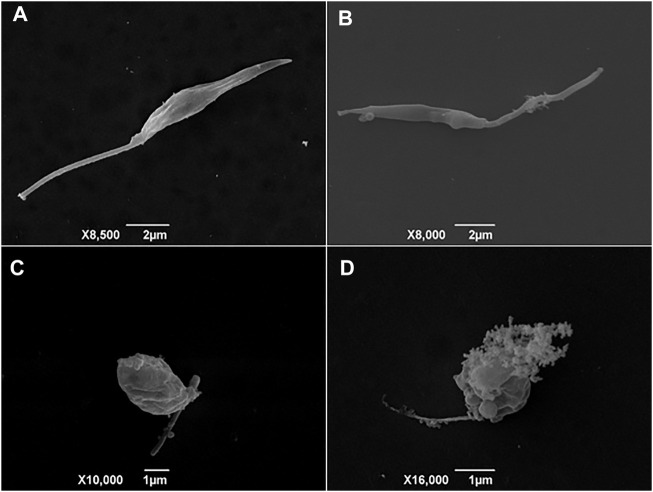
Scanning electron microscopy (SEM) analysis of promastigotes of *L. amazonensis* incubated with BA5. **(A)** Untreated control cells with normal morphology, **(B)** parasites treated with IC_50_/2 value of BA5, **(C)** parasites treated with IC_50_ value of BA5, **(D)** parasites treated with twice the IC_50_.

After determining the activity against promastigotes and amastigotes forms of *Leishmania sp*., assays were performed to elucidate a possible mechanism of action of the BA5. First, ultrastructural analysis by scanning electron microscopy (SEM) was used to evaluate the morphology of *L. amazonensis* promastigotes treated or not with BA5. Untreated promastigotes had the typical elongated shape of the parasite without visible alterations in the plasma membrane or in cell volume (Figure 3A). In contrast, parasites treated for 48 h with BA5 (2.2, 4.5 or 9.0 µM) presented several morphological alterations, such as membrane protrusions resembling surface blebs (Figure 3B), flagella damage, increase in size (Figure 3C), and body deformation (Figure 3D).

### BA5 Induces Apoptosis Like-Death in *L. amazonensis* Promastigotes

**FIGURE 4:**
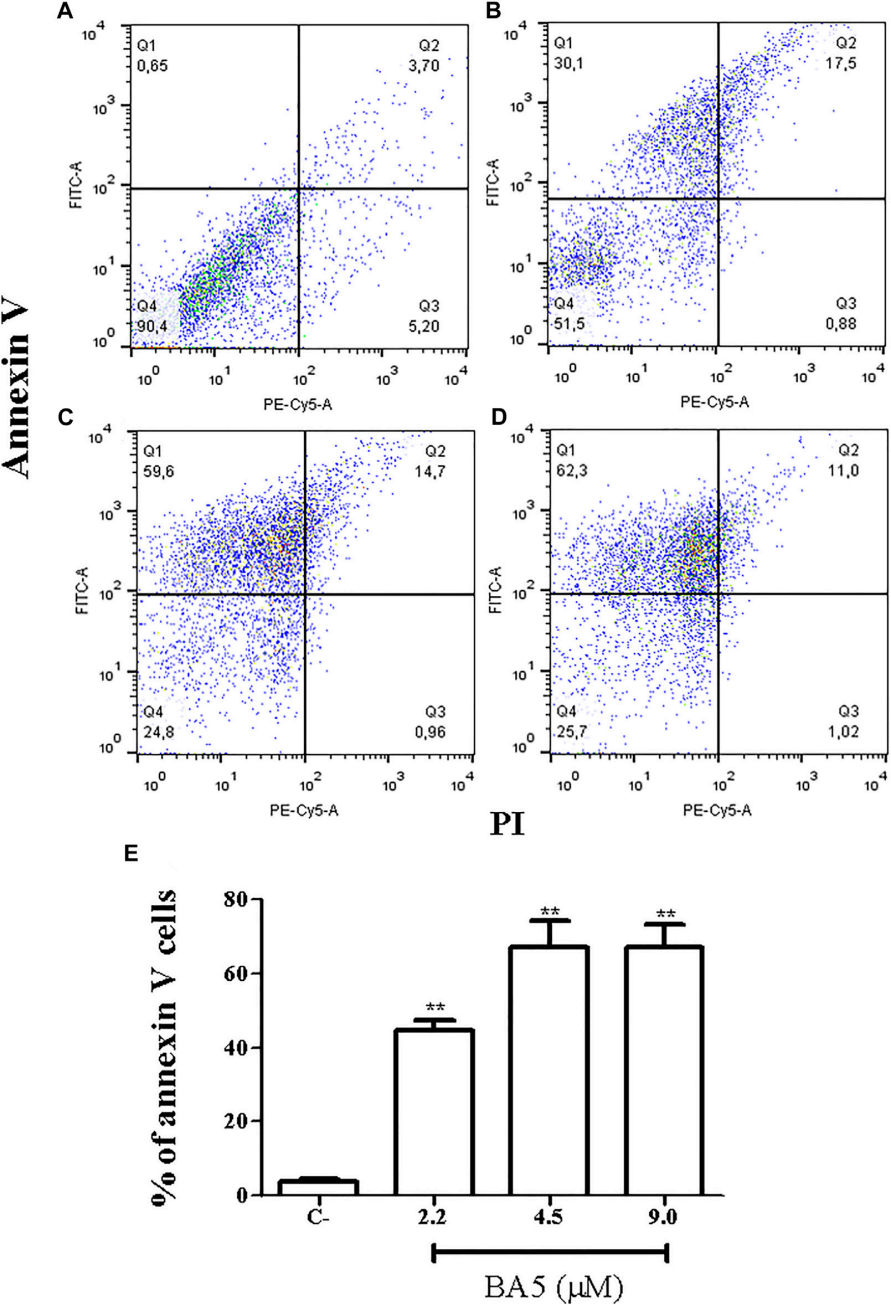
Flow cytometry analysis of cell death pattern. *L. amazonensis* promastigotes were treated with BA5 and incubated with propidium iodide (PI) and annexin V after 48 h of incubation. **(A)** Untreated promastigotes **(B)** promastigotes treated with 2.2 µM of BA5 **(C)** promastigotes treated with 4.5 µM of BA5 **(D)** promastigotes treated with 9.0 µM of BA5 **(E)** Percentage of stained cells for annexin V after 48 h of treatment with BA5. Values represent the means ± S.E.M. of three determinations obtained in one of two experiments performed. **p < 0.01 compared to stimulated and untreated cells.

Because the formation of blebbing in the membrane, cell rounding, and flagella damage generally culminates in the formation of apoptotic bodies (Basmaciyan and Casanova, 2019), we evaluated the mechanism by which compound BA5 could cause parasite death. Promastigotes were double-stained with Annexin-V-FITC and propidium iodide (PI) for flow cytometry analysis. Untreated cells were Annexin-V and PI-negative, demonstrating cell viability. The percentage of promastigotes positive only for annexin-V was 27.3% after treatment with IC_50_/2 value of BA5, 51.25% when cells were treated with the IC_50_ value of BA5 and 54.45% when cells were treated with 2x IC_50_ of BA5. This data suggests that these cells were in early stages of apoptosis-like death. No significant difference in the number of necrotic cells was observed after treatment when compared to untreated controls (Figure 4).

### BA5 Acts Independently of Mitochondrial Membrane Depolarization

**FIGURE 5:**
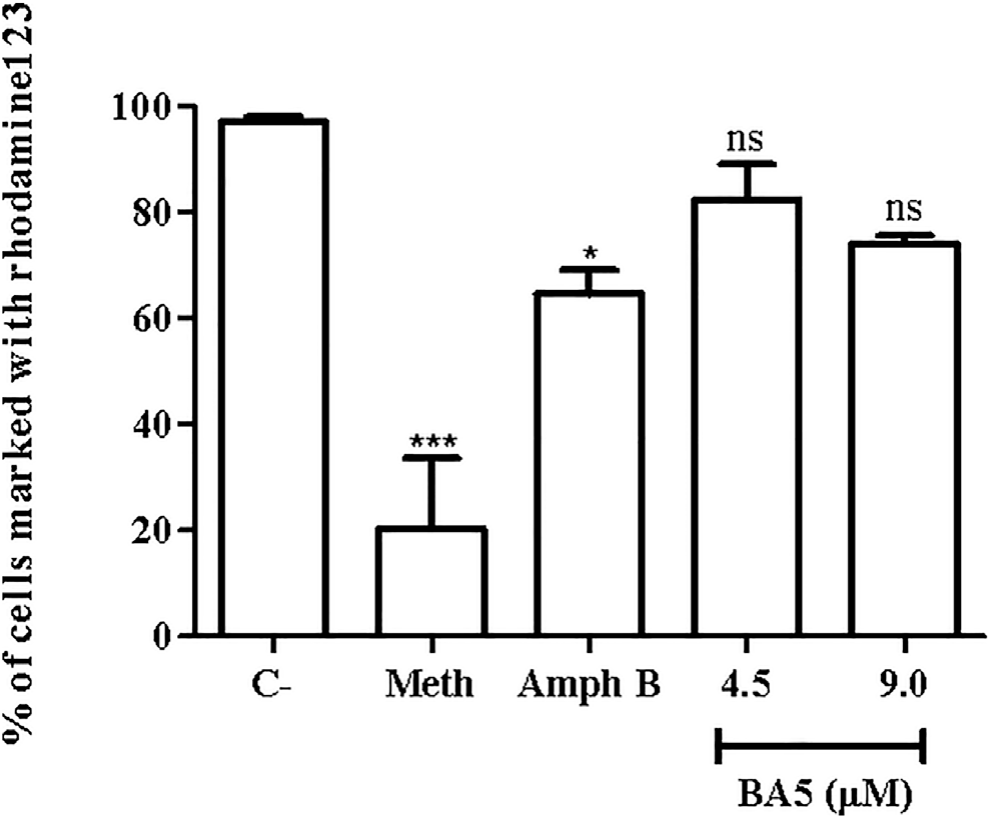
Mitochondrial membrane potential of *L. amazonensis* promastigotes incubated with BA5. Promastigotes were incubated or not with BA5 at concentrations of IC_50_ and 2x IC_50_ and amphotericin B (IC_50_). Methanol (Meth) was used as positive control. After 72 h of incubation, parasites were marked with rhodamine123. The samples were acquired in a LSRFortessa flow cytometer and analyzed by FlowJo software (50,000 events were collected and analyzed). ***p < 0.001, compared to untreated group.

To better understand the pathways that lead to apoptosis-like death, the mitochondria potential of *L. amazonensis* was evaluated by flow cytometry, after BA5 treatment and incubation with rhodamine123. As shown in Figure 5, the intensity of rhodamine123 was not significantly altered by incubation with BA5 at IC_50_ and 2xIC_50_ values of the drug. Amphotericin B and methanol, two known drugs able to induce mitochondrial alterations, reduced the intensity of the rhodamine 123.

### BA5 Induces Cell Cycle Arrest in *L. amazonensis* Promastigotes

**FIGURE 6:**
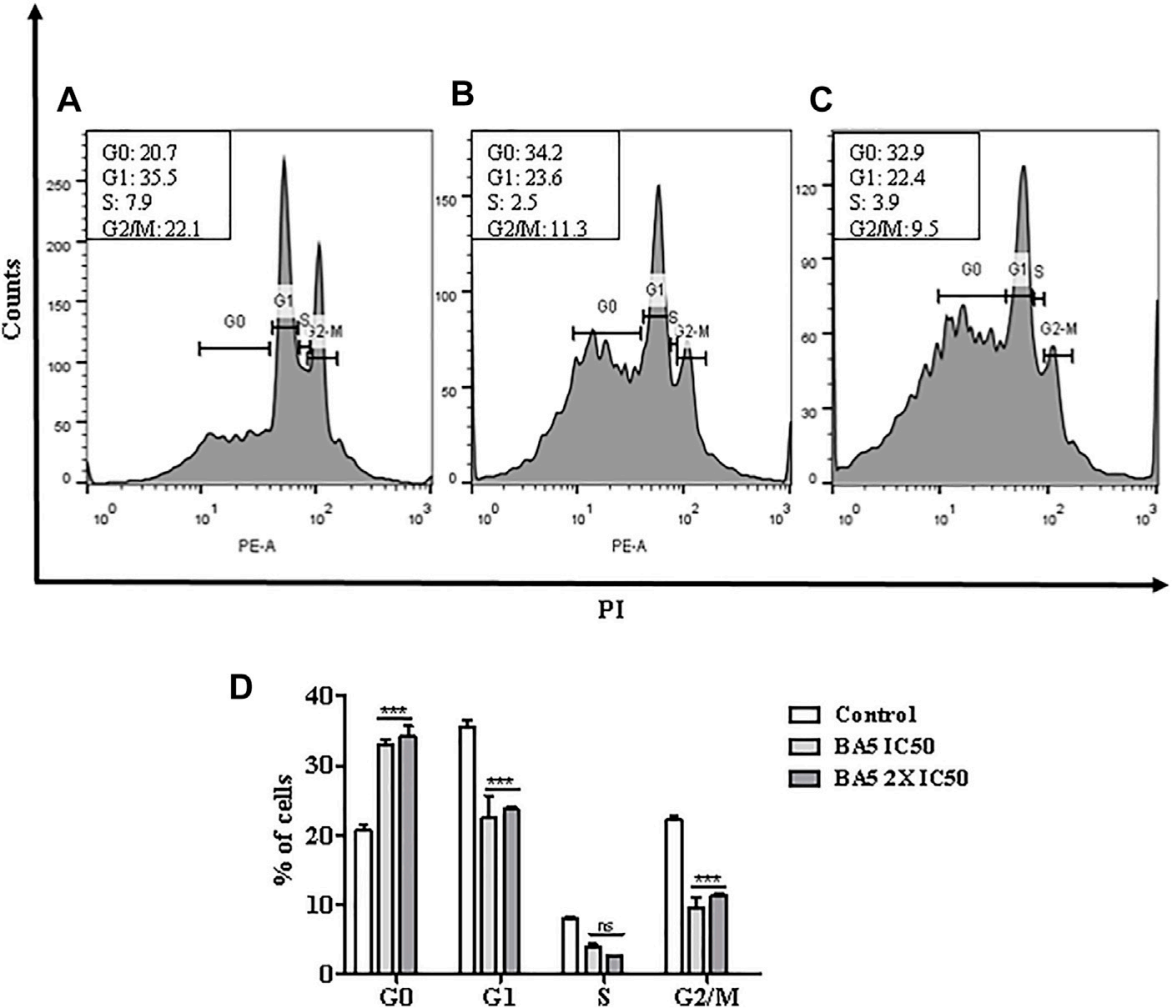
Analysis of cell cycle progression after treatment with BA5 using propidium iodide by flow cytometry. The distribution and percentage of parasites in pre-phase G0, G1, S and G2/M phase of the cell cycle are indicated. Cells treated with IC_50_ value **(B)**, and 2x IC_50_ value **(C)** concentrations of BA5, compared to untreated control **(A)**. **(D)** Percentage of cells in different phases of cell cycle. Values represent the means ± S.E.M. of three determinations obtained in one of two experiments performed. ***p < 0.001 compared to stimulated and untreated cells.

Next, flow cytometric analysis after cell permeabilization and labelling with PI was used for quantification of nuclear DNA of parasites. Promastigotes of *L. amazonensis* treated with BA5 and amphotericin B with IC_50_ and 2xIC_50_ values were marked with PI and analyzed by flow cytometry. Figure 6 shows the distribution of cellular DNA through the cell cycle of the parasites in the absence and presence of the tested compounds. A significant increase in population of cells in pre-phase G0 and a significant decrease in population of cells in G2/M were observed in cells treated with IC_50_ value (Figure 6B), and 2x IC_50_ value (Figure 6C) concentrations of BA5, compared to untreated control (Figure 6A), 24 h after incubation.

### Synergistic Effects of BA5 and Amphotericin B

**FIGURE 7:**
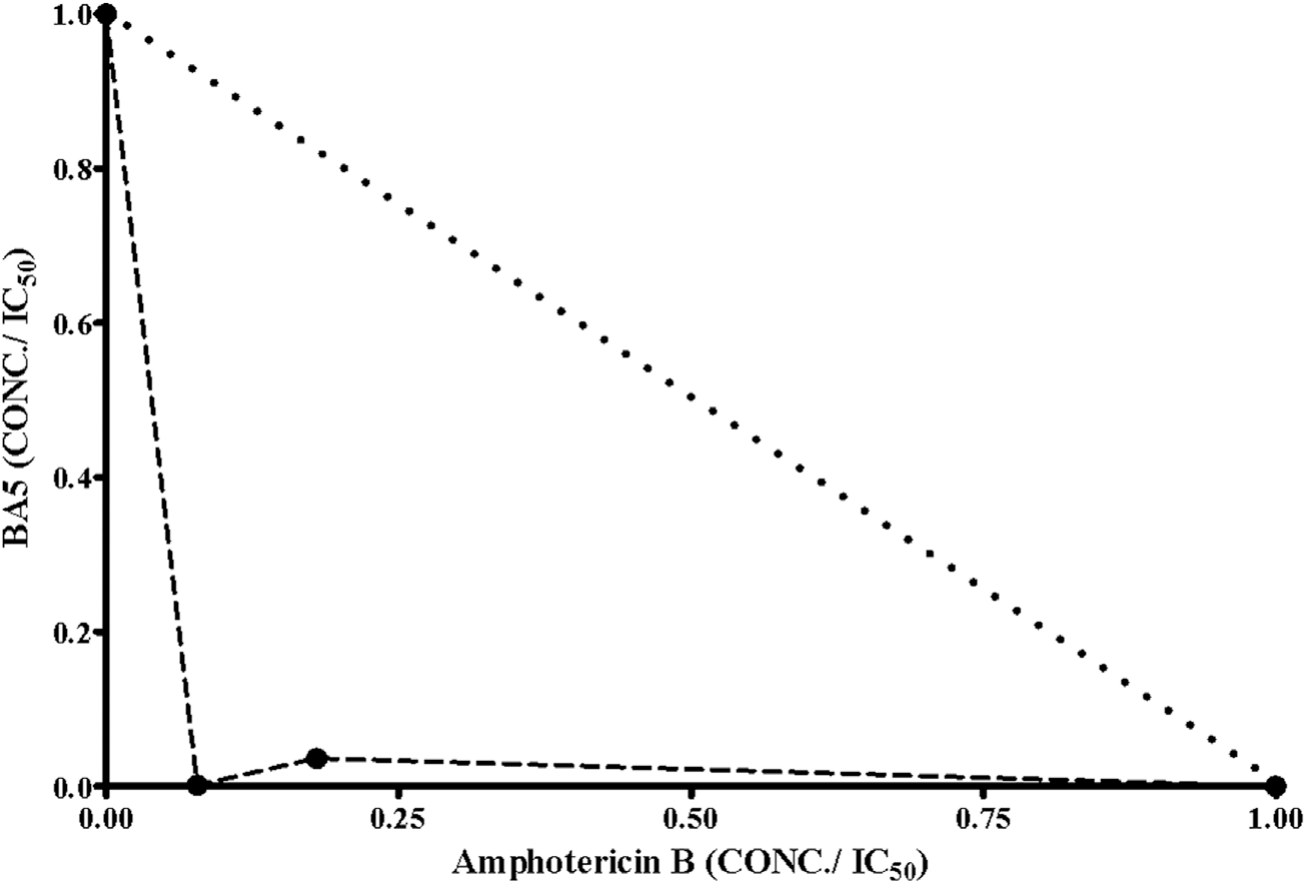
Isobologram showing the synergistic effects between BA5 and amphotericin B on *L. amazonensis* promastigotes. Broken lines correspond to the predicted positions of the experimental points for additive effects.

The antileishmanial effect of BA5 and amphotericin B combination was investigated on promastigote forms of *L. amazonensis*. The combination of the drugs reduced the IC_50_ values of amphotericin B by seven times and decreased the IC_50_ values of BA5 by 50 times compared to each drug separately. The combination index values (0.15 ± 0.09 µM) Table 3 associated with a concave isobologram revealed that BA5 and amphotericin B have synergistic effects (Figure 7).

**TABLE 3:** Concentration reductions and combination rates by BA5 and amphotericin B on *L. amazonensis* promastigotes.

Compounds	IC_50_ ± S.D. (µM)^a^		FIC**	CI***
	Drug alone	Combination		
BA5	4.50 ± 1.1	0.09 ± 0.01	0.018	0.15 ± 0.09
Amphotericin B	0.09 ± 0.02	0.012 ± 0.006	0.129	—

a IC_50_ values were calculated using quadruplicate concentrations and two independent experiments were performed: **Fractional inhibitory concentrations (FIC). *** Combination index (CI). Cut: CI, value of 0.1–0.7, synergism; 0.7–0.85, moderate synergism; 0.85–0.9, slight synergism; 0.9–1.1, additivity; > 1.1, antagonism. S.D., standard deviation.

## Discussion

The search for molecules of natural origin has intensified and played an important role in the development of new drugs (Newman and Cragg, 2016). Betulinic acid is a molecule in the class of lupane-type pentacyclic triterpenes found in all parts of higher plants. This molecule has a vast number of activities described in the literature, such as antitumor, antimalarial, anti-HIV, analgesic, anti-inflammatory, and bactericidal (Fujioka et al., 1994; Kim et al., 2001; Ali-Seyed et al., 2016; Li et al., 2017). Previous studies have shown that substitution on the carboxyl group can generate more potent molecules than the prototype, aiming at different pharmacological targets (Krogh et al., 1999; Chandramu et al., 2003; Barbosa Filho et al., 2004; Mullauer et al., 2010; Halder et al., 2018; Mehrizi et al., 2018). A recent work from our group reported that the insertion of amines at C-28 in BA increased the anti-*T. cruzi* activity by inducing ultrastructural changes in the parasite (Meira et al., 2016). This is the first report, however, regarding the contribution of the incorporation of amides on C-28 as drug design strategy to enhance the antileishmanial activity.

Structural changes in betulinic acid prototype are associated with reduction in the cytotoxicity of the new compounds (Alakurtti et al., 2010; Newman and Cragg, 2016; Mehrizi et al., 2019). In agreement with these studies, we observed that the BA5 derivative is less cytotoxic (CC_50_ = 31.1 µM) than their prototype (CC_50_ = 18.8 µM). Furthermore, BA5 was less cytotoxic than amphotericin B (CC_50_ = 3.3 µM). These data reinforce the importance of structural chemical modifications in reducing cytotoxicity and enhancing the practical applicability of the compounds in medicinal chemistry.

Previous reports showed the activity of betulinic acid derivatives against *Leishmania sp* promastigotes (Magaraci et al., 2003; Dominguez-Carmona et al., 2010). Heterocyclic derivatives of betulinic acid showed activity against *L. donovani* with IC_50_ values of 8.9–30 µM and carbamate derivatives against *L. infantum* with IC_50_ values of 25.8 µM (Alakurtti et al., 2010; Sousa et al., 2014). Dominguez-Carmona et al., 2010, reported activities of an acetate derivative against *L. amazonensis* (IC_50_ = 44.9 µM). In our study, the addition of amines in the C-28 of BA5 optimized the effects of the molecule in relation to the prototype and showed better antileishmanial activity than other triterpenes.

BA5 was able to inhibit macrophage infection and the number of intracellular forms of *L. amazonensis* with a reduced IC_50_ value (4.1 ± 0.7). Other studies demonstrated that treatment with alkaloid derivatives of the betulinic acid reduced in 83% the number of infected macrophages by *L. amazonensis*, using higher drug concentrations, with an IC_50_ value of 210 µM (Moraes et al., 2015). Furthermore, incubation with nanoparticle-loaded betulinic acid reduced the number of macrophages infected by *L. major* (81%), improving its activity and reducing toxic effects (Mehrizi et al., 2019).

It is suggested that the higher SI, more effective and safer a drug would be during *in vivo* treatment (Pritchett et al., 2014). In this study, the SI value of amphotericin was higher than BA5. Amphotericin B presents an elevated cost, high toxicity and its use requires hospitalization of patients. This drug present difficult structural changes in the liposomal molecule to reduce toxicity (Filippin and Souza, 2006), whereas BA5 is a prototype for the design and its selectivity can be increased with conformational alterations (Meira et al., 2016).

Apoptosis is an important event in the context of the host’s immune response and in the successful establishment of infection by leishmania. The survival of these parasites within macrophages is a crucial issue in the pathogenesis of the disease in the mammalian host (Aliança et al., 2017). Despite being an event markedly of multicellular organisms, currently, there are studies in the literature that suggest a mechanism similar to apoptosis in single-celled eukaryotes. In trypanosomatids, regulated cell death is shown to be advantageous to prevent the activation of the immune system and, therefore, the survival of intracellular parasites (Baréa et al., 2018). Flow cytometry analysis demonstrated that BA5 acts to induce cell death by apoptosis in parasites. These data were confirmed when we evaluated the morphology of the parasites treated with BA5. Similar morphological changes, such as flagellar damage, appearance of blebs and increase in the size of *L. amazonensis*, have previously been associated with induced apoptotic death. Some changes were seen in the same species of parasites treated with a series of triazine hybrids and with a calpain inhibitor (Marinõ et al., 2014; Baréa et al., 2018). In addition, the treatment of promastigotes of *L. amazonensis* with BA5 induce changes in the cell cycle of the parasites with arrested in the G0/G1 phase and a significant decrease in population of cells in G2/M (Meira et al., 2017). Altogether, these results suggest that BA5-induced apoptosis may have led to DNA degradation.

The mitochondria play an important role in cell death by apoptosis (Koonin and Aravind, 2002). Rhodamine 123 is a cationic lipophilic dye that is readily sequestered by active mitochondria without cytotoxic effects. Additionally, this dye can be used to assay mitochondrial membrane potential in populations of apoptotic cells (Mehta and Shaha, 2004; Marinho et al., 2014). Reports indicate that the mitochondria integrity is a good indicator of structural changes in the kinetoplastid parasite (Silva et al., 2020). To elucidate the mechanism of cell death possibly induced by BA5, we evaluated the potential of mitochondrial membrane. BA5 did not induce alterations in membrane potential in *L. amazonensis* promastigotes, suggesting that the action of the compound is independent of this pathway. Moreover, Basmaciyan and Casanova, 2019 demonstrated that mitochondrial depolarization when preceded by transient hyperpolarization in leishmania, and the loss of plasma membrane integrity is not specific apoptosis markers.

Drug combination is an alternative applied in the clinic for treatment of leishmaniasis that have advantages over current monotherapy. Amphotericin B is a second-choice drug for the treatment of leishmaniasis in many places around the world. Combined drug therapy can be an important tool for reducing toxic effects, as well as reducing the duration of treatment and improving treatment compliance by the patient (Bastos et al., 2016; Roatt et al., 2020). In this regard, some studies were caried out with the aim to associating promising compounds to amphotericin B (Marinho et al., 2014; Mehrizi et al., 2019; Roatt et al., 2020; Silva et al., 2020). In this work, BA5 was shown to increase the activity of the reference drug (CI = 0.15; synergistic action), showing a promising profile for drug combination. This result encourages further investigations since the combination of drugs is becoming increasingly attractive to combat parasitic diseases. This semi-synthetic derivative was able to prevent the parasite development and invasion into host cells during *T. cruzi* infection, with synergistic activity when used in combination to benznidazole (Meira et al., 2016). Our research group also reported the activity of BA5 increasing the immunosuppressive effect of dexamethasone. BA5 presented synergistic effects with dexamethasone on the inhibition of lymphocyte proliferation, suggesting a promising profile for drug combination therapeutic schemes (Meira et al., 2017).

In conclusion, this work showed that BA5 has antileishmanial activity against different species causative of cutaneous and visceral leishmaniasis. BA5 presents low cytotoxicity, *in vitro* activity against parasite proliferation and macrophage infection by leishmania*.* Although its mechanism of action still needs further evaluation, it was found that BA5 promotes cell death due to apoptosis and arrest the cell cycle progression*.* Therefore, BA5 may be a suitable candidate for antileishmanial drug development, alone or in combination with other drugs.

## Data Availability

The original contributions presented in the study are included in the article/Supplementary Material, further inquiries can be directed to the corresponding author.
